# Involvement of 5-Lipoxygenase in the Corticosteroid-Dependent Amyloid Beta Formation: In Vitro and In Vivo Evidence

**DOI:** 10.1371/journal.pone.0015163

**Published:** 2011-01-06

**Authors:** Simone Puccio, Jin Chu, Domenico Praticò

**Affiliations:** Department of Pharmacology, Temple University School of Medicine, Philadelphia, Pennsylvania, United States of America; Biological Research Center of the Hungarian Academy of Sciences, Hungary

## Abstract

**Background:**

Numerous studies show that high circulating level of glucocorticosteroids is a biochemical characteristic of Alzheimer's disease (AD). These stress hormones can increase the amount of AD-like pathology in animal models of the disease. Since they also up-regulate the 5-Lipoxygenase (5-LO), an enzyme which modulates amyloid beta (Aβ) formation, in the present paper we tested the hypothesis that this enzymatic pathway is involved in the glucocorticoid-induced pro-amyloidotic effect.

**Methodology/Principal Findings:**

Incubation of neuronal cells with dexamethasone resulted in a significant increase in 5-LO activity and Aβ formation. By contrast, pharmacological inhibition of 5-LO prevented the dexamethasone-dependent increase in Aβ levels. Mouse embryonic fibroblasts responded with a significant increase in Aβ formation after dexamethasone challenge. However, this effect was abolished when dexamethasone was incubated with fibroblasts genetically deficient for 5-LO. No difference in the glucocorticoid receptor levels was observed between the two groups. Finally, treatment of wild type mice with dexamethasone resulted in a significant increase in endogenous brain Aβ levels, which was prevented in mice genetically lacking 5-LO.

**Conclusions:**

These findings suggest that 5-LO plays a functional role in the glucocorticoid-induced brain AD-like amyloid pathology.

## Introduction

Alzheimer's disease (AD) is the most common form of neurodegenerative disease with dementia in the elderly, affecting approximately 6–8% all persons aged >65 years [1]. While only a minority of AD cases is caused by missense mutations in genes for either the Aβ precursor protein (APP) or Presenilin-1 and -2, the cause of sporadic AD remains unclear, and a combination of environmental and genetic factors with epigenetic events has been implicated [2]. Psychosocial stress has been suggested to be one important environmental factor that can influence AD age of onset and/or development [3]. Several clinical studies have linked dysregulation of stress hormone levels, such as glucocorticoids, with AD pathogenesis. Plasma cortisol levels are increased in subjects with mild cognitive impairment and in AD patients [4]–[6]. Recently, it has been demonstrated that chronic stress and glucocorticoids promote amyloid beta (Aβ) deposition and tau accumulation in transgenic mouse models of AD [7], [8]. Among the different biological actions, dexamethasone is known to increase the expression levels of the 5-Lipoxygenase (5-LO), an enzyme widely expressed in the central nervous system (CNS) where it localizes mainly in neuronal cells [9]. Previous studies have reported that 5-LO immunoreactivity is increased in hippocampi of AD patients, and that its protein levels are higher in cortex and hippocampus, but not cerebellum, of AD brains when compared with healthy controls [10], [11]. Further, genetic absence of 5-LO results in a significant reduction of brain Aβ levels and deposition in a transgenic AD mouse model, suggesting that this enzymatic pathway plays a functional role in modulating the amyloidotic phenotype of this model [11]. In the present study, we sought to determine whether 5-LO was involved in the glucocorticoid-dependent Aβ elevation. To this end, we investigated the effect of dexamethasone on Aβ formation and metabolism in the presence and in the absence of 5-LO enzymatic activity in vitro and in vivo. Here we confirm that glucocorticoid challenge enhances the synthesis of Aβ, and report the novel finding that pharmacological blockade or genetic absence of 5-LO prevents this biological effect. Our findings underscore a new mechanism by which psychological stress affects AD-like amyloid pathology and suggest that 5-LO could be a therapeutic target in individuals where stress management or pharmacological reduction of glucocorticoids approaches are not applicable.

## Materials and Methods

### Cell lines, cell culture and treatment

Neuro-2 A neuroblastoma (N2A) cells stably expressing human APP carrying the K670 N, M671L Swedish mutation (APPswe) were grown in Dulbecco's modified Eagle medium supplemented with 10% fetal bovine serum, 100 U/mL streptomycin (Cellgro, Herdon, VA, USA) and 400 µg/mL G418 (Invitrogen, Carlsbad, CA,USA), at 37°C in the presence of 5% CO_2_. HEK293-C99 cells stably transfected with human C-terminal fragment C99 of human APP containing Aβ sequence were kindly provided by Dr. Robert W. Doms (University of Pennsylvania, Philadelphia, USA) and kept in culture as previously described [11]. For each experiment, equal numbers of cells were plated in six-well plates, 24 hr later media were removed and fresh media containing either dexamethasone (1 mM stock solution dissolved in H_2_O; cat. # D2915. Sigma-Aldrich, St. Louis, MO) or vehicle were added. After an overnight incubation, supernatants were collected for biochemistry assays, and cell pellets harvested for biochemical analyses.

### Mouse Embryonic Fibroblasts isolation

Mouse embryonic fibroblasts (MEFs) were obtained as previously described [11]. Briefly, pregnant female mice were sacrificed 12–13 days after observing vaginal plugs. Uterine horns were dissected out, briefly rinsed in 70% ethanol and placed into a sterile Petri dish containing phosphate buffered saline (PBS) without bivalent cations. Each embryo was isolated and separated from its placenta and surrounding membranes. The head, liver and kidneys were removed and used for genotyping, and a cell suspension of the remaining tissue was prepared by trituration in 1 ml trypsin-EDTA. Following gentle shaking at 37°C for 15 min with 100 Kunitz units/mL of DNAse, the resulting cell suspensions were incubated on ice for additional 15 min, then subjected to low speed centrifugation (500 g) for 5 min. The resulting cell pellet was plated into a 10 cm dish with pre-warmed MEF medium (DMEM with high glucose, 10% fetal bovine serum, L-glutamine (200 mM), and penicillin/streptomycin). The culture medium was replaced with fresh DMEM after 24 h. After 2–3 passages, MEFs were genotyped again to avoid possible errors in initial genotyping due to maternal tissue contamination. APP x 5LO^+/+^ and APP x 5LO^−/−^ MEFs were always used for the experiments described in the present paper.

### Mice and treatments

All experiments were performed in accordance with animal protocols approved by the Institutional Animal Care and Usage Committee, and the U.S. National Institute of Health guidelines (approval ID 3311). All mice used in the present study were females. Twelve-month-old 5-LO^−/−^ mice and wild type (WT) littermates were administered with an intra-peritoneal injection of either dexamethasone (dissolved in PBS at 1 mg/ml) or PBS alone for 7 days. Dexamethasone was administered at 1 mg/kg body weight or PBS vehicle. 24 h after the final injection, animals were killed following procedures recommended by Panel or Euthanasia of the American Veterinarian Medical Association. Brains were removed after perfusion and immediately stored at −80°C for biochemical analyses.

### Biochemical analyses

All of the biochemical analyses were always performed in triplicate and in a coded fashion.

### Aβ sandwich ELISA assay

Aliquots of brain samples were homogenized and extracted in 0.2% diethylamine (DEA)/50 mM NaCl at 1∶10 W/v. Brain homogenates were centrifuged for 1 hr at 100,000× g at 4°C, and supernatants were neutralized to pH 8.0 with 1∶10 v/v of 0.5 Tris-HCl/pH 6.8. Protein concentration in DEA extracts was determined using the BCA Protein Assay kit (Pierce, Rockford, IL). The Aβ1-40 and 1-42 levels in the supernatants and in mouse brain homogenates were determined using specific sandwich ELISA kits as previously described, and following the manufacturer's instructions [11], [12] (Wako Chemicals, Japan).

### Leukotriene B4 ELISA assay

Leukotriene B4 levels were assayed in supernatants and brain homogenates by a specific and sensitive immunoassay according to the manufacturer's recommendation (Assay Designs; Ann Arbor, MI, USA), and as previously described [13].

### Western blot analyses

Cells were washed with PBS and lysed in RIPA buffer (50 nmol/L Tris-HCL, 150 mmol/L NaCl, 1% Nonidet P-40, 0.5% sodium deoxycholate, 2 mmol/L EDTA, 0,1% sodium dodecyl sulfate) in the presence of an EDTA-free protease inhibitor cocktail tablet (Roche Applied Science). Cell lysates were sonicated and centrifuged at 14,000 g for 20 min at 4°C. Aliquots of brain homogenates and cell lysates were assayed for protein concentration by a BCA Protein Assay Kit. Equal amount of protein from cell lysates or brain extracts (30 µg) were electrophoresed on 3–8 % Tris-acetate gels and 10% Bis-Tris gels, according to the molecular weight of the target molecule, and then transferred on nitrocellulose membranes (Bio-Rad, Richmond, CA, USA). They were blocked with Odyssey blocking buffer for 1 h; and then incubated with primary antibodies overnight at 4°C, as previously described [11]–[13]. After three washings with T-TBS, incubation with IRDye secondary antibodies (LI-COR Bioscience, NE, USA) were performed at 22°C for 1 h. The membranes were developed using infrared fluorescence detection on the Odyssey and Aerius infrared imaging systems (LI-COR Bioscience, USA). Antibodies and dilutions used for western blot analysis were as follows: anti-APP N-terminal raised against amino acids 66–81 for total APP (22C11; 1∶1500; Chemicon Inter., USA), anti-BACE1 (1;200; IBL America), anti-ADAM-10 (1: 500 dilution; Chemicon), anti-PS1 (1: 200; Cell Signaling, USA), anti-nicastrin (1: 200; Cell Signaling), anti-PEN2 (1: 200; Invitrogen, USA), anti-APH 1 (1: 200; Millipore, USA); anti-5LO (clone 33;1∶500; BD Bioscience), anti-β actin (1;4000, Santa Cruz Biotechnology), anti-Glucocorticoid Receptor (1∶500 Pierce Biotechnology). IRDye infrared secondary antibodies were from LI-COR Bioscience.

### Immunofluorescence microscopy

Cell immunostaining was carried out as previously described [12]. Briefly, N2A-APPswe cells were plated on glass cover slips and the following day fixed in 4% paraformaldehyde in PBS for 15 min at 22°C. After rinsing several times with PBS, cells were incubated in a blocking solution (5% normal serum / 0.4 % TX-100) for 1 h at 22°C and then with the primary antibody against 5-LO (1∶500; BD Bioscience) overnight at 4°C. After several washings with PBS, cells were incubated for 1 h with a secondary Alexa546- conjugated antibody (1∶800; Invitrogen). Cover slips were mounted using VECTASHIELD mounting medium (Vector Laboratories, Burlingame, CA) and analyzed with an Olympus BX60 fluorescent microscope (Olympus, Center Valley, PA). Excitation at 405 nm for 4′,6′-diamidino-2-phenylindole and 543 for Alexa546 was generated by diode and He-Ne ion lasers, respectively. Fluorescence emission was collected at 425–475 nm for 4′, 6-diamidino-2-phenylindole and 555–656 nm for Alexa546. Olympus Fluo BView 1.3 (Center Valley, PA, USA) was used for image acquisition. Control cover slips were processed as described above except that no primary antibody was added to the solution.

### Statistical analysis

Data are presented as the mean ± S.E.M. For each experimental setting, data are expressed as percentage of the control value of that specific experiment. Each control was arbitrarily set at 100%. The percentage values obtained from different experiments were then averaged and plotted as percentage of control. At least three independent experiments were always performed in each condition. An effect of treatment was defined as significant if p<0.05 by analysis of variance (ANOVA), and subsequently by student unpaired two-tailed *t*-test.

## Results

### Dexamethasone increases Aβ formation in vitro via a 5-LO-dependent manner

**Figure 1:**
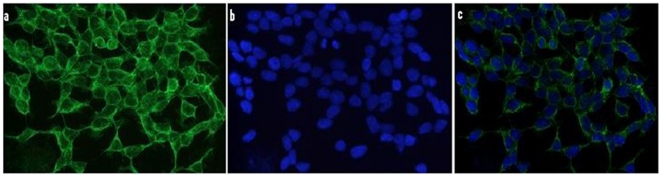
Immunocytochemical localization of 5-LO in N2A-APPswe cells. Cells were plated on glass cover slips, fixed in 4% paraformaldehyde, then blocked with serum, incubated with a primary antibody against 5-LO. (a) 5-LO immunofluorescence, (b) DAPI staining, (c) overlap of the two images. (magnification × 40).

**Figure 2:**
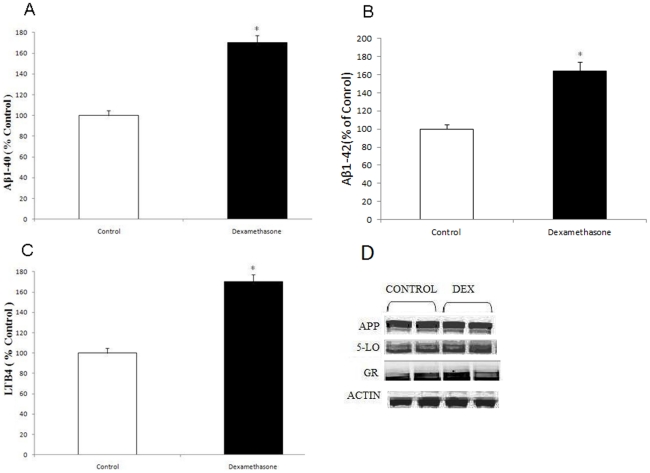
Dexamethasone increases Aβ formation in neuronal cells. N2A-APPswe cells were incubated with Dexamethasone (closed bars) (10 µM) or vehicle (open bars) and conditioned media collected and assayed for (A) Aβ 1–40 (B) Aβ1-42 levels (n = 6 per condition, *p<0.001), and (C) LTB4 (*p<0.05). D. Representative immunoblots of cell lysates probed with antibodies against APP, 5-LO, and glucocorticoid receptor (GR) in control and after dexamethasone treatment. Values represent mean ± SEM.

First, by using immunofluorescence microscopy we investigated the expression levels of 5-LO in the neuronal cells, N2A, which will be used in our paper. As shown in Figure 1, we found that these cells express high levels of this protein with the most intense positive immunoreaction having a diffuse pattern and mainly localized in the cytosol. Next, we challenged the N2A cells with dexamethasone (10 µM) or vehicle, and observed the effect on Aβ formation and 5-LO activation. Compared with vehicle, dexamethasone induced a significant increase in Aβ 1–40 and 1–42 levels (Fig. 2A,B), which was associated with a significant elevation in the production of leukotriene B4 (LTB4), the major metabolic product of 5-LO activation (Fig. 2C). Under this experimental conditions steady state expression levels of 5-LO and GR receptor were unchanged (Fig. 2D).

Since we observed that dexamethasone incubation resulted in 5-LO activation, as demonstrated by the increase in LTB4 formation, next we tested the hypothesis of whether pharmacological blockade of 5-LO will modulate the dexamethasone-dependent increase of Aβ formation. To this end, we used two different pharmacological approaches: a selective and direct 5-LO inhibitor (i.e., AA-861) [14], and a specific blocker of the 5-LO activating protein (i.e., MK-591), also known as FLAP, which is necessary for a full 5-LO activity [14]. At the concentration used AA-861 significantly reduced LTB4 formation (control: 96±7; AA-861 1 µM: 55±2; AA-861 5 µM: 27±1.5; AA-861 10 µM: 8.6±1.5 pg/ml). A similar effect was observed in samples treated with MK-591 (control: 99±5.4; MK-591 5 µM: 54±3.3; MK-591 10 µM: 23±2.7; MK-591 25 µM: 7.36±1.8 pg/ml).

**Figure 3:**
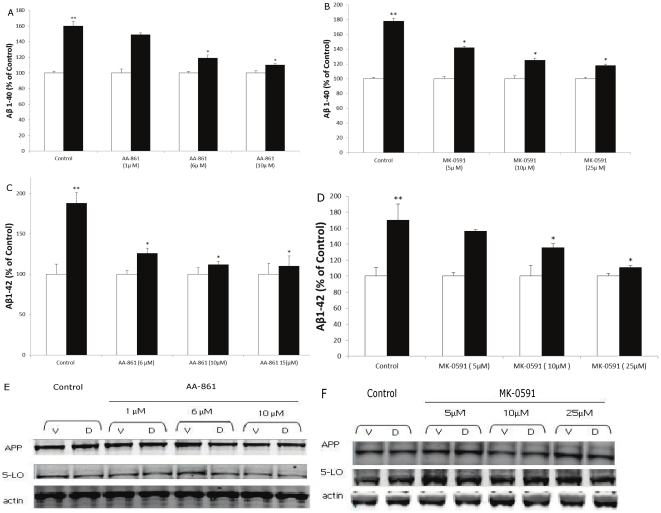
Pharmacological blockade of 5-LO activation prevents dexamethasone-induced Aβ formation. N2A-APPswe cells were incubated with different concentrations of AA-861 (closed bars), and MK-0591 (closed bars), or vehicle (open bars) overnight and then treated with dexamethasone (10 µM) for 6 h. A–D. Conditioned media collected and Aβ1–40 and Aβ 1–42 levels assayed (n = 3 per condition, **p<0.01; *p<0.05). Values represent mean ± SEM. E, F. Representative western blot images of APP and 5-LO protein expression after AA-861 and MK-0591 treatments.

In both cases, the drugs dose-dependently prevented the dexamethasone-dependent effect on Aβ 1–40 and 1–42 formation (Fig. 3A–D). This effect was independent from any influence on the steady state levels of APP or 5-LO in the same cells (Fig. 3E,F).

### Effect of dexamethasone on Aβ formation in MEF-5-LO+/+ or MEF-5-LO−/−

**Figure 4:**
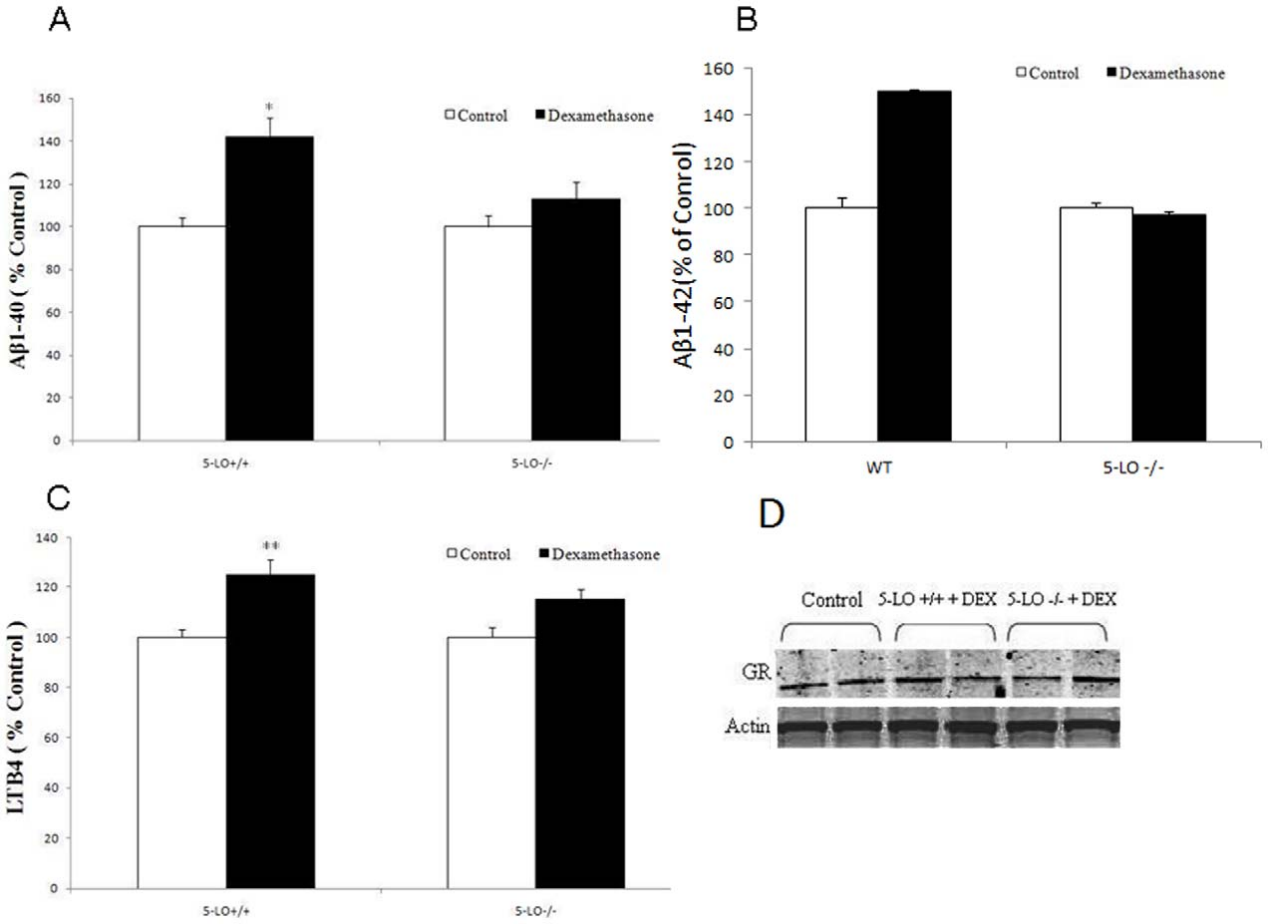
Effect of dexamethasone on Aβ formation in MEF-APP 5-LO+/+ or MEF-APP 5-LO−/−. MEF were incubated with dexamethasone (10 µM) and conditioned media collected and assayed for (A) Aβ1-40 and (B) Aβ1-42 levels (n = 6 per condition, *p<0.001), and (C) LTB4 (**p<0.05). D. Representative immunoblots of cell lysates probed with antibodies against glucocorticoid receptor (GR) and β-actin. Values represent mean ±SEM.

To further substantiate the involvement of 5-LO in the dexamethasone-dependent effect on Aβ formation, we next used MEFs isolated from transgenic mice over-expressing the Swedish APP mutant (i.e., Tg2576) [15], or Tg2576 genetically deficient for 5-LO (Tg2576/5-LO^−/−^) [11], and incubated them with dexamethasone. As shown in Figure 4A and B, we found that only cells with the 5-LO available but not the ones lacking the enzyme responded to dexamethasone with a significant increase in Aβ 1–40 and 1–42 formation, which was associated with a similar elevation in LTB4 levels (Fig. 4C). Immunoblot analyses did not show any significant difference in the expression levels of the GR receptor between the cells with and without the 5-LO gene and in the presence or absence of dexamethasone (Fig. 4D)

### Dexamethasone increases Aβ levels in vivo via a 5-LO-dependent manner

**Figure 5:**
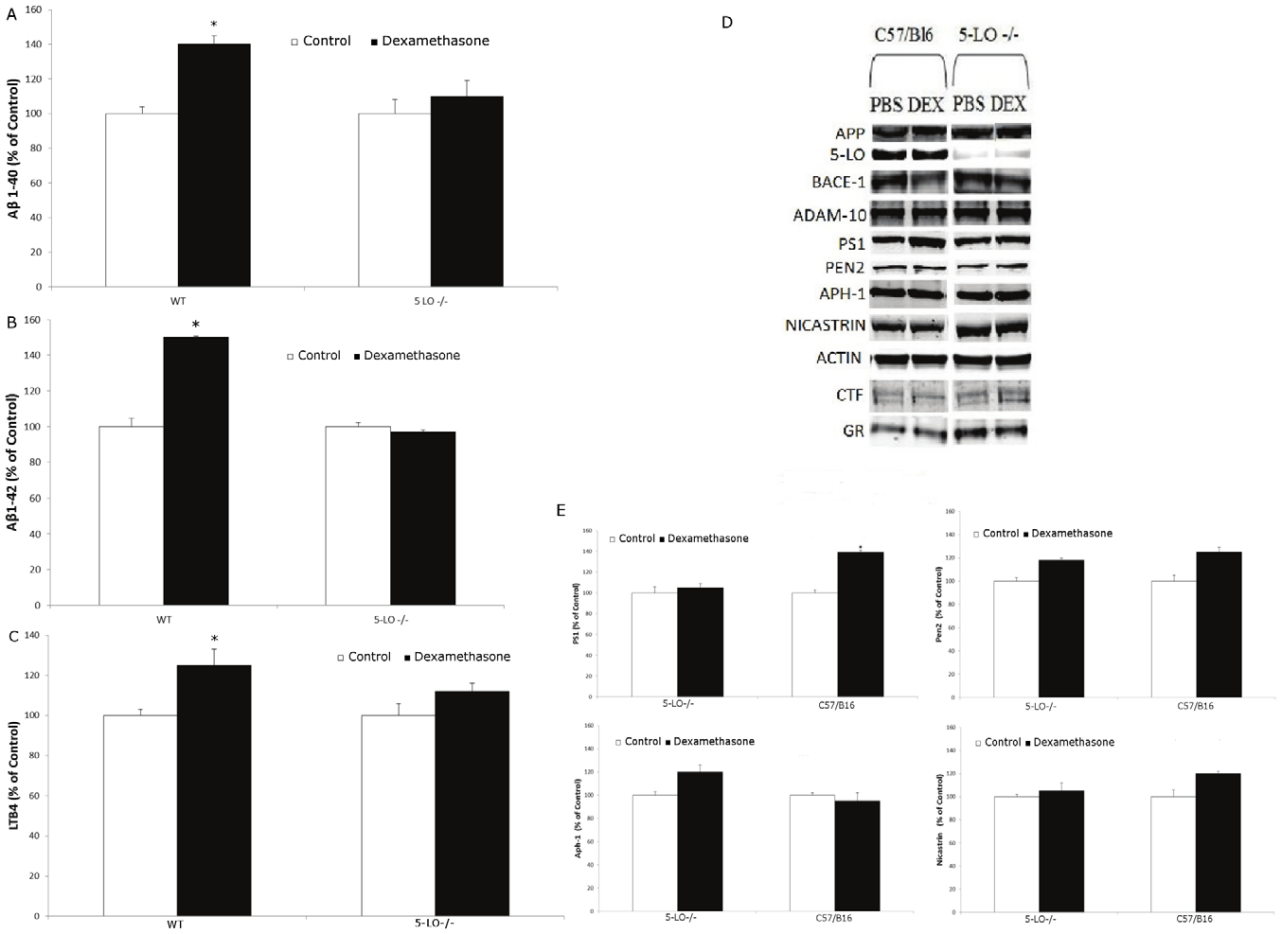
Dexamethasone increases Aβ levels in vivo. 5-LO knock-out (5-LO−/−) mice and wild type littermates (WT) were treated daily for 7 d with dexamethasone 5 mg/kg i.p. (n = 4) or PBS (n = 3). Brain homogenates were assayed for total (A) Aβ 1–40 and (B) Aβ1-42 levels (open bars: PBS, closed bars; dexamethasone, *p<0.05). C. LTB4 levels in brain homogenates from mice treated with dexamethasone (closed bars, n = 4) or PBS (open bars, n = 3). D. Representative immunoblots of brain homogenates probed with antibodies against APP, 5-LO, BACE-1, ADAM-10, Nicastrin, PS1, APH-1, Pen-2 and glucocorticoid receptor (GR). E. Quantification of protein blots normalized to β-Actin as a loading control (*p<0.05). Values represent mean ± SEM.

To confirm that the effect of dexamethasone on Aβ formation requires the availability of 5-LO also in vivo, next 5-LO^−/−^ and wild type littermates were administered with dexamethasone for one week. At the end of the treatment animals were sacrificed and their brains assayed for endogenous Aβ levels. In accordance with the *in vitro* effect, we observed that dexamethasone induced a significant increase in the endogenous levels of Aβ in the brains of WT mice (Fig. 5A, B). However, this effect was abolished in the brains of mice genetically deficient for 5-LO (Fig. 5A, B). Dexamethsaone treatment resulted also in a statistically significant increase in the LTB4 levels in brains form C57Bl6 mice but not 5-LO−/− mice (Fig. 5C). Western blot analysis showed that the two groups of animals had similar expression levels of the GR receptor, and that these levels were not influenced by the dexamethasone treatment (Fig. 5D). Furthermore, no significant difference was observed for steady state levels of APP, BACE-1, ADAM-10 between the two groups of mice and after the dexamethasone challenge. By contrast, we observed that PS1 levels, one of the major components of the γ-secretase complex, were significantly elevated in WT mice treated with dexamethasone, but not in 5-LO−/− mice (Fig. 5E).

### Dexamethasone effect on Aβ formation via 5-LO is γ-secretase dependent

**Figure 6:**
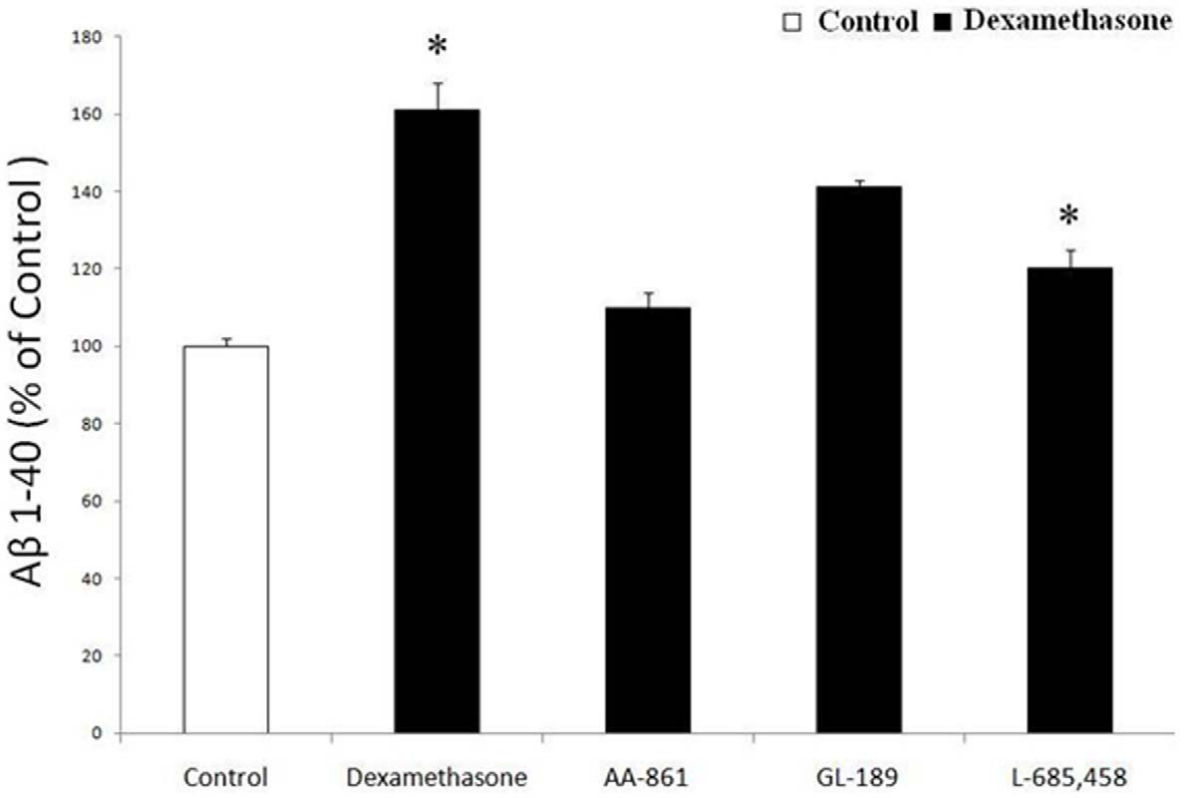
Dexamethasone effect on Aβ formation is γ-secretase-dependent. HEK-C99 cells were incubated with dexamethasone (10 µM) in the presence of a 5-LO inhibitor (AA-861 10 µM), a specific β-secretase inhibitor (GL-189, 0.5 µM) or a γ-secretase inhibitor (L-685,458, 1 µM), and conditioned media assayed for Aβ1- 40 levels. Values represent mean ± SEM of 3 experiments (*p<0.05).

Since the in vivo study indicated that dexamethsone increases Aβ formation by influencing the γ-secretase pathway, next we tested the hypothesis that dexamethasone by activating 5-LO affects Aβ formation directly via this pathway. To this end we used HEK293 cells stably expressing human APP C99, the direct substrate for γ-secretase. Incubation of these cells with dexamethasone (10 µM) induced a significant increase in Aβ levels (Fig. 6), which was associated with a significant increase in LTB4 (54.3±5.8 *vs* 99.3±pg/ml, p<0.05). However, when these cells were incubated with AA-861, the effect of dexamethasone on Aβ formation was blunted (Fig. 6). On the other hand, under the same conditions GL189 (EMD Biosciences Inc, La Jolla, CA), a BACE-1 inhibitor [16], did not induce any change in Aβ secretion. By contrast, L-685,458, a specific and potent γ-secretase inhibitor [17], completely prevented the dexamethasone-dependent effect on Aβ in these cells (Fig. 6).

## Discussion

The present paper shows for the first time that the 5-LO enzymatic pathway is involved in the dexamethasone–dependent increase of Aβ formation in vitro and in vivo. The enzyme 5-LO is a member of a large family of non-heme iron dioxygenases, whose main action is to oxidize and convert fatty acids such as arachidonate to 5-hydroxy-peroxy-eicosatetraenoic (5-HPETE) and 5-hydroxy-eicosatetraenoic acids (5-HETE), which are then metabolized into different leukotrienes [18]. This enzyme is widely expressed in the CNS, where it localizes mainly in neuronal cells. Its presence has been documented in various brain regions where significant changes are associated with aging [13], [19]. Since aging is one of the strongest risk factors for developing sporadic AD, it has been suggested that this pathway may be involved in its pathogenesis [20]. While the cause of sporadic AD remains unknown, a combination of environmental and genetic factors has been implicated in its pathogenesis. Psychosocial stress leading to altered levels of stress hormones, i.e., glucocorticoids, is considered one important environmental risk factor that can influence AD age of onset and/or development [21]. However, the mechanism(s) by which corticosteroids accelerate the pathogenesis of AD remain to be fully elucidated [22]. Interestingly, in vitro and in vivo studies showed that dexamethasone increases the expression levels of 5-LO [23], suggesting a possible biological link between stress hormones and the development of AD. With the present paper we provide the first biological evidence that 5-LO is an essential step in the dexamethasone-dependent Aβ formation. First, we showed that in neuronal cells dexamethasone increases Aβ formation and activates the 5-LO enzymatic pathway. The requirement of 5-LO in the dexamethasone-dependent Aβ formation was initially supported by a pharmacological approach. Thus, by using two distinct pharmacologic tools which block 5-LO activation we prevented the effect of dexamethasone on Aβ. The specific role and functional importance of 5-LO in this phenomenon was further corroborated when we used cells genetically deficient for this enzyme. In this setting, while MEFs with the 5-LO+/+ responded with an elevation in Aβ after the dexamethasone challenge, the same cells but without the 5-LO, i.e., 5-LO^−/−^, did not. Taken together these observations establish the 5-LO as a novel and important biological link between dexamethasone and Aβ formation. In an effort to elucidate potential mechanisms by which activation of 5-LO by dexamethasone would ultimately result in an elevation of Aβ levels, we first of all checked whether there was an effect on total levels of APP, the precursor of the Aβ. Under our experimental conditions no effect on APP was observed. Next, since dexamethasone biological activity is regulated by the presence of its own receptor, i.e., GR, we wanted to assess whether these levels were altered by the pharmacological tools used. However, no significant change in the protein levels of this receptor was observed. Importantly we also did not observed any difference in the GR levels in cells with and without the gene for the 5-LO, supporting the concept that the lack of effect of the dexamethasone in the 5-LO ^−/−^ cells was not secondary to lower levels or absence of the GR.

In the final part of our studies, we sought to investigate whether the findings obtained in the in vitro models were reproducible also in vivo. Thus, when we injected with dexamethasone WT mice we observed a significant increase in 5-LO activation and Aβ formation, which were completely abolished in mice that were genetically deficient for the 5-LO. Since previous work indicated that 5-LO alters Aβ formation by modulating the γ-secretase pathway of the APP processing [11], we wanted to see whether this was also the case in our in vivo experimental setting. To this end, we observed that while the steady state levels of APP, BACE-1 and ADAM-10 were unchanged, PS1 protein levels, a major component of the γ-secretase complex, were significantly increased in the brains of mice receiving dexamethasone, suggesting an involvement of this secretase. This observation was further corroborated by another set of experiments, where we used HEK293 cells that stably express APP-C99, the precursor of Aβ and the immediate substrate for the γ-secretase. In these cells, pharmacological inhibition of 5-LO activation prevented the dexamethasone-dependent increase of Aβ formation to a similar extent of a classical γ-secretase inhibitor. Taken together these data support the concept that dexamethasone activates the 5-LO enzymatic pathway, which then affects the APP processing via modulation of the γ-secreatse pathway and ultimately the Aβ levels.

Our findings have particular relevance to AD because it is established that AD patients display elevated circulating cortisol levels [24]. Clinical data also suggest that a stressful lifestyle, which would result in elevated cortisol levels can be a risk factor for AD onset [25]. In addition, another strong risk factor for AD is the presence of the apoE4 allele, which has been shown to elevate CSF cortisol levels [26] more so than the E3 or the E2 allele.

In conclusion, our studies unveil for the first time a novel functional role for the 5-LO enzymatic pathway in the dexamethasone-dependent elevation of Aβ, since its pharmacological inhibition and genetic absence prevent it. Our findings underscore a novel mechanism by which corticosteroids may affect AD-like amyloid pathology and suggest that 5-LO could be a therapeutic target in individuals where stress management or pharmacological reduction of glucocorticoids approach is not applicable.
